# ROCK-ALS: Protocol for a Randomized, Placebo-Controlled, Double-Blind Phase IIa Trial of Safety, Tolerability and Efficacy of the Rho Kinase (ROCK) Inhibitor Fasudil in Amyotrophic Lateral Sclerosis

**DOI:** 10.3389/fneur.2019.00293

**Published:** 2019-03-27

**Authors:** Paul Lingor, Markus Weber, William Camu, Tim Friede, Reinhard Hilgers, Andreas Leha, Christoph Neuwirth, René Günther, Michael Benatar, Magdalena Kuzma-Kozakiewicz, Helen Bidner, Christiane Blankenstein, Roberto Frontini, Albert Ludolph, Jan C. Koch, The ROCK-ALS Investigators, Shahram Attarian

**Affiliations:** Author Affiliations: Reference center for neuromuscular disorders and ALS, University Hospital la Timone, Marseille, France; Department of Neurology, University Medical Center Göttingen, Germany; Institute for Sleep Medicine and Neuromuscular Disorders, Muenster University Hospital, Münster, Germany; Neuromuscular Diseases Unit/ALS Clinic, Kantonsspital St. Gallen, St. Gallen Switzerland; Reference Center for ALS and other Motoneuron Disorders, University Hospital Bretonneau, Tours, France; Department of Neurology, Technical University of Munich, Munich, Germany; Department of Neurology, Technical University of Munich, Munich, Germany; Department of Neurology, Alfried Krupp Hospital, Essen, Germany; Department of Neurology, Jena University Hospital, Jena, Germany; Department of Neurology, Technical University of Dresden, Dresden, Germany; German Center for Neurodegenerative Diseases (DZNE) Dresden, Dresden, Germany; Department of Neurology, University Medical Center Göttingen, Germany; Department of Neurology, University Medical Center Göttingen, Germany; Department of Neurology, University Medical Center Göttingen, Germany; Center for ALS and other motor neuron disorders, Charité–Universitätsmedizin Berlin, Germany; Department of Neurology, Hannover Medical School, Hannover, Germany; Pharmacy at the University of Leipzig Medical Center, Leipzig, Germany; Department of Neurology, University Medical Center Göttingen, Germany; Department of Neurology, University of Ulm, Ulm, Germany; Center for ALS, University Hospital Pasteur, CHU de Nice, France; University of Kansas Medical Center, Kansas City, KS, United States; Department of Neurology, University of Ulm, Ulm, Germany; Department of Neurology, University of Würzburg, Würzburg, Germany; Department of Neurology, University Medical Center Göttingen, Germany.; ^1^Department of Neurology, Technical University of Munich, Munich, Germany; ^2^Department of Neurology, University Medical Center Göttingen, Göttingen, Germany; ^3^Neuromuscular Diseases Unit/ALS Clinic, Kantonsspital St. Gallen, St., Gallen, Switzerland; ^4^Reference Center for ALS and Other Rare Motoneuron Disorders, University Hospital Gui de Chauliac, Montpellier, France; ^5^Department of Medical Statistics, University Medical Center Göttingen, Göttingen, Germany; ^6^Department of Neurology, Technical University of Dresden, Dresden, Germany; ^7^German Center for Neurodegenerative Diseases (DZNE) Dresden, Dresden, Germany; ^8^Department of Neurology, University of Miami, Miami, FL, United States; ^9^Department of Neurology, Medical University of Warsaw, Warsaw, Poland; ^10^Münchner Studienzentrum, Technical University of Munich, Munich, Germany; ^11^Pharmacy at the University of Leipzig Medical Center, Leipzig, Germany; ^12^Department of Neurology, University of Ulm, Ulm, Germany

**Keywords:** amyotrophic lateral sclerosis, disease-modification, clinical trial protocol, ROCK inhibition, study design

## Abstract

**Objectives:** Disease-modifying therapies for amyotrophic lateral sclerosis (ALS) are still not satisfactory. The Rho kinase (ROCK) inhibitor fasudil has demonstrated beneficial effects in cell culture and animal models of ALS. For many years, fasudil has been approved in Japan for the treatment of vasospasm in patients with subarachnoid hemorrhage with a favorable safety profile. Here we describe a clinical trial protocol to repurpose fasudil as a disease-modifying therapy for ALS patients.

**Methods:** ROCK-ALS is a multicenter, double-blind, randomized, placebo-controlled phase IIa trial of fasudil in ALS patients (EudraCT: 2017-003676-31, NCT: 03792490). Safety and tolerability are the primary endpoints. Efficacy is a secondary endpoint and will be assessed by the change in ALSFRS-R, ALSAQ-5, slow vital capacity (SVC), ECAS, and the motor unit number index (MUNIX), as well as survival. Efficacy measures will be assessed before (baseline) and immediately after the infusion therapy as well as on days 90 and 180. Patients will receive a daily dose of either 30 or 60 mg fasudil, or placebo in two intravenous applications for a total of 20 days. Regular assessments of safety will be performed throughout the treatment period, and in the follow-up period until day 180. Additionally, we will collect biological fluids to assess target engagement and evaluate potential biomarkers for disease progression. A total of 120 patients with probable or definite ALS (revised El Escorial criteria) and within 6–18 months of the onset of weakness shall be included in 16 centers in Germany, Switzerland and France.

**Results and conclusions:** The ROCK-ALS trial is a phase IIa trial to evaluate the ROCK-inhibitor fasudil in early-stage ALS-patients that started patient recruitment in 2019.

## Background and Preclinical Data

Up to now only riluzole and edaravone have been approved as disease-modifying treatments for amyotrophic lateral sclerosis (ALS), but their efficacy is limited. Thus, there is an urgent need to identify more efficient disease-modifying drugs to improve function and prolong survival for this devastating motoneuron disorder.

Rho kinase (ROCK) has recently emerged as a novel therapeutic target for neurodegenerative disorders [reviewed in (1)]. ROCK is a serine/threonine kinase with two isoforms: while ROCK1 is expressed preferentially in peripheral tissue, ROCK2 is highly expressed in the central nervous system (CNS). Axonal growth inhibitory molecules (e.g., Nogo, MAG, OMgp, ephrins, semaphorins) bind to specific extracellular receptors and signal via ROCK to trigger axonal degeneration, growth cone collapse, and impaired axonal regeneration. In non-neuronal structures, regulation of the actin cytoskeleton plasticity by ROCK also mediates vasoconstriction and vascular remodeling (2). Interestingly, ROCK also regulates cell survival via Akt and mTOR (3–5).

Levels of ROCK increase with age and tissue of ALS patients show increased levels of ROCK2 as well as its downstream targets LIMK1 and cofilin (6, 7). Moreover, in the SOD1 (G93A) mouse model of ALS, increased ROCK activity results in higher levels of phosphorylated adducin as well as activation of phosphatase and tensin homolog (PTEN) and decreased Akt activity, suggesting a deactivation of these cell survival pathways (5). These findings validate the known effects of ROCK activation on pathways regulating the plasticity of the actin cytoskeleton and neuronal survival. Adducin is phosphorylated by ROCK and promotes the interaction between actin and spectrin resulting in actin bundling and stabilization of the cytoskeleton (8). PTEN activation by ROCK is important for its effects on neuronal survival. Since PTEN inhibits phosphatidylinositol (3,4,5) tris-phosphate, which is a central activator of Akt/PKB signaling, PTEN activation by ROCK also exerts negative effects on cell growth, proliferation and metabolism. Via this pathway active ROCK not only to inhibits Akt but also the mammalian target of rapamycin (mTOR) complex 1, which is also an important positive regulator of protein synthesis, cell growth and regeneration (9).

ROCK can be inhibited by fasudil, an isochinoline derivative that was originally developed as a vasodilatory drug and licensed in Japan in 1995 for the treatment of vasospasm following subarachnoid hemorrhage (SAH). Several thousands of patients have been treated with fasudil since. Fasudil has also been tested in numerous clinical trials for other applications, most frequently in cardiovascular disease, such as angina pectoris, Raynaud's syndrome, pulmonary hypertension and arterial hypertension (2). In the CNS, the effects of fasudil were assessed in a phase III trial in patients with acute ischemic stroke, where fasudil treatment significantly improved clinical outcome (10). Significant amounts of fasudil are taken up in the brain (11). Although only intravenous formulations of fasudil are licensed in Japan and China, oral (tablet) formulations, including extended release capsules, were used in clinical trials in humans. The longest published exposure to fasudil in humans is 8 and 12 weeks for angina pectoris and pulmonary arterial hypertension, respectively. Side effects included allergic skin reactions, a slight drop in systolic blood pressure and reversible renal impairment without major safety concerns arguing against longer dosing as required for the treatment of ALS (12, 13). Because of ample clinical experience and a well-known safety profile, fasudil represents an excellent candidate for repurposing as a disease-modifying therapy in ALS. Most importantly, in the SOD1 (G93A) mouse model of ALS, fasudil prolonged survival and improved motor function, which was independently reproduced by two groups (5, 14, 15). Fasudil also improved the regenerative response in the neuromuscular junction, which was accompanied by a modulation of microglial activity. ROCK inhibition not only influenced the morphology of microglia, but also attenuated the lipopolysaccharide-induced release of chemokines and cytokines (14, 16). Other groups showed beneficial effects in the SMN1 knockdown model of spinal muscular atrophy (17). These preclinical data, therefore, suggests ROCK as a novel drugable target with disease-modifying effects in ALS.

## Methods, Design and Analysis

### Objectives

The primary objective of this phase IIa study (proof-of-concept study) is to evaluate the safety and tolerability of intravenous fasudil in two different doses. Participants will be treated for 20 days and followed up for a period of 6 months. Secondary objectives include the survival time and the change of revised ALS Functional Rating Scale (ALSFRS-R), ALS Assessment Questionnaire (ALSAQ-5), Edinburgh Cognitive and Behavioral ALS Screen (ECAS), Motor Unit Number Index (MUNIX) and slow vital capacity (SVC) from baseline to 4 weeks, 3 months, and 6 months after treatment initiation. A further secondary aim is to determine safety and tolerability until the end of the 4 weeks infusion period.

### Study Design

**Figure 1:**
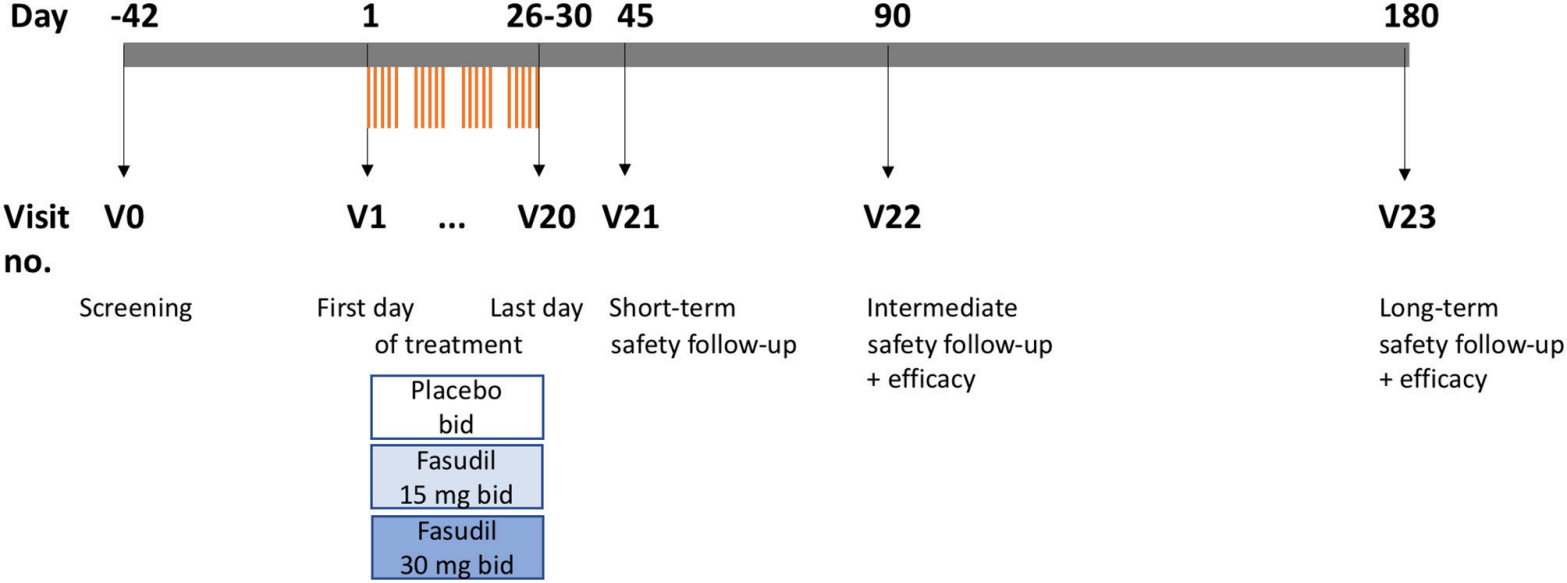
Scheme of trial (drawn to time scale). V, visit; bid, two times daily.

ROCK-ALS is a multi-center, international, randomized, double-blind, placebo-controlled, prospective, dose-finding Phase IIa trial. During its design we considered the “Guideline on clinical investigation of medicinal products for the treatment of amyotrophic lateral sclerosis” of the European Medicines Agency (EMA) where appropriate and the protocol adheres to the “Standard Protocol Items for Randomized Trials” (SPIRIT). It has been registered with the European Clinical Trials Database (Eudra-CT, https://eudract.ema.europa.eu/) under the number 2017-003676-31, with the German Clinical Trials Register (DRKS) under the number DRKS00013948, and on clinicaltrials.gov (NCT03792490). Figure 1 shows the design of the trial.

### Participants

#### Inclusion and Exclusion Criteria

Patients with clinical probable, probable laboratory supported or definite ALS and a disease duration of more than 6 and less than 18 months (disease onset defined as date of first muscle weakness, excluding fasciculations and cramps) are eligible for participation in the ROCK-ALS trial. A co-medication with riluzole is compulsory. A previously started treatment with edaravone should be continued throughout the trial. The slow vital capacity must be at least 65% of normal. Main exclusion criteria are previous tracheostomy, continuous assisted ventilation, gastrostomy, severe arterial hypotension, and liver or kidney insufficiency. Importantly, there should be no personal and family history of intracerebral aneurysms or Moyamoya disease due to a theoretical risk of intracranial hemorrhage associated with fasudil treatment. The full list of inclusion and exclusion criteria is depicted in Table 1.

**Table 1:** Inclusion and exclusion criteria.

**Inclusion criteria**	**Exclusion criteria**
•Probable (clinically or laboratory) or definite ALS according to the revised version of the El Escorial World Federation of Neurology criteria •Disease duration more than 6 months and less than 18 months (inclusive). Disease onset defined as date of first muscle weakness, excluding fasciculations and cramps •Vital capacity more than 65% of normal (SVC; best of three measurements) •Age: ≥18 years •Patients have to be treated with Riluzole (2 × 50 mg/d), must be stable for at least 4 weeks before randomization •Patients who have started on Edaravone therapy shall continue Edaravone treatment. Edaravone treatment must not be discontinued for reasons of trial participation. •Women of childbearing age must be non-lactating and surgically sterile or using a highly effective method of birth control and have a negative pregnancy test. Acceptable methods of birth control with a low failure rate i.e., <1% per year) when used consistently and correct are such as implants, injectables, combined oral contraceptives, hormonal intrauterine devices (IUDs), sexual abstinence or vasectomized partner •Capable of thoroughly understanding all information given and giving full informed consent according to GCP •Patients have to have a valid health insurance, when recruited in a center in France	•Previous participation in another clinical study involving trial medication within the preceding 12 weeks or five terminal half times of the longest to be eliminated trial medications (whichever is longer) or previous participation in this trial •Tracheostomy or continuous assisted ventilation of any type during the preceding 3 months before randomization or a significant pulmonary disorder not attributed to ALS, which may complicate the evaluation of respiratory function, intermittent non-invasive ventilation is permitted. •Patients with a history of intracranial bleeding, known intracerebral aneurysms or Moyamoya disease, or positive family history for the above. If only family history positive, MR- or X-ray-based cranial imaging not older than 24 months must confirm absence of bleeding, aneurysms or Moyamoya. •Gastrostomy •Any medical condition known to have an association with motor neuron dysfunction or involving neuromuscular weakness or another neurodegenerative disease, e.g., PD or AD, which might confound or obscure the diagnosis of ALS •Presence of any concomitant life-threatening disease or impairment likely to interfere with functional assessment •Patients with known arterial hypotension (resting blood pressure <90/60 mmHg) or previous hypotensive episodes or requiring treatment for increasing of blood pressure, such as fludrocortisone, midodrine, etilefrine, cafedrine, or theodrenaline •Patients with an uncontrollable or unstable arterial hypertensive disease (resting blood pressure >180 mmHg systolic and/or >120 mmHg diastolic under current antihypertensive medication) •Known pulmonary hypertension and any medication prescribed for treatment of pulmonary hypertension •Confirmed hepatic insufficiency or abnormal liver function (stable ASAT and/or ALAT >3 times the upper limit of the normal range) and determined to be non-transient through repeat testing •Renal insufficiency with a glomerular filtration rate (GFR) <60 ml/min/1.73 m^2^ (calculated by MDRD equation) and determined to be non-transient through repeat testing •Major psychiatric disorder, significant cognitive impairment or clinically evident dementia precluding evaluation of symptoms •Hypersensitivity to any component of the study drug •Liable to be not cooperative or comply with the trial requirements (as assessed by the investigator), or unable to be reached in the case of emergency •Pregnant or breast-feeding females or females with childbearing potential, if no adequate contraceptive measures are used •Prisoners or subjects who are involuntary incarcerated •Patients subject to legal protection measures

#### Recruitment

Patients will be recruited and assessed for eligibility by the ALS outpatient clinics of the highly specialized academic centers taking part in the study, where several hundred ALS patients are seen each year on average. Referring centers and established neurologists will be informed about the ROCK-ALS trial by direct contact and information letters. Patient information sheets and information leaflets for medical professionals (primary care physicians and neurologists) will be issued and distributed to national patient organizations of the participating countries which will be asked to make the information available on their websites. A website for the ROCK-ALS trial has been launched to deliver information for patients and medical professionals (http://rock-als.uni-goettingen.de).

### Randomization, Stratification and Blinding

This trial has three parallel groups: fasudil 15 mg twice daily, fasudil 30 mg twice daily, and matching placebo. Patients are randomized to treatment using an allocation ratio of 1:1:1. The randomization list will be centrally generated using a computerized system stratified by geographical region and type of onset (bulbar, spinal). Patients will be assigned to the bulbar or spinal stratum according to the location of the earliest experienced ALS symptom (defined by the first muscle weakness or in the case of bulbar onset, by the presence of dysarthria and/or dysphagia). In the case of a patient with simultaneous onset of spinal and bulbar symptoms, onset will be defined as bulbar. Cervical and respiratory onsets are stratified to the spinal onset stratum. The randomization list will be generated by the Biometry and Bioinformatics Core using a pseudo-random number generator.

This study includes double-blind (participant- and investigator blind) treatment. Study medication will be packed and blinded by the Pharmacy at University of Leipzig Medical Center, Germany, according to the randomization list. Each patient medication package will be sent together with the sealed unblinding codes to the sites. The investigator at the site will take care that each patient is provided with the study medication box of the correct randomization number. The randomization list will be kept in safe and confidential custody at Pharmacy at University of Leipzig Medical Center, Germany. For all patients, emergency codes will be available to the investigator. A code, which reveals the treatment group for a specific study patient, may be opened during the study only if the choice of treatment depends on the study subject's therapy assignment. During the study, emergency unblinding should occur only by accessing the study patient's emergency code.

### Drug Procurement and Intervention

Fasudil hydrochloride hydrate will be delivered as medicinal product to the Pharmacy at University of Leipzig Medical Center, Germany, and the trial medication as well as the according placebo will be produced, packaged, stored, blinded, labeled, and shipped to the trial sites in accordance with the regulations of the participating country and good manufacturing practice (GMP) (Annex 13 of the EU Guideline for GMP).

Trial medication will be supplied as fasudil hydrochloride hydrate solution 30 mg for IV application (2 × 1 ml of 15 mg/ml fasudil hydrochloride hydrate), or fasudil hydrochloride hydrate solution 15 mg for IV application (1 ml of 15 mg/ml fasudil hydrochloride hydrate and 1 ml NaCl 0.9%). The appropriate Placebo to fasudil hydrochloride solution will be supplied as 2 × 1 ml NaCl 0.9%. Placebo solution is identical in appearance to the study medication (clear, colorless fluid), but does not contain the active ingredient.

The trial drug or placebo has to be diluted in 100 ml NaCl 0.9% prior to administration and is administered two times daily IV as infusion over 45 min using a CE-certified infusion pump. The second application starts 7 ± 1 h after the start of the first application.

### Study Procedures

The trial is structured into 24 visits (V0–V23). V0 is the screening visit and may take place up to 42 days before the first treatment. Treatments are then performed twice daily from V1 to V20 on work days, excluding week-ends and holidays. Subsequently, trial participants are seen at three follow-up visits, V21, V22, and V23, at 45, 90, and 180 days after baseline, respectively. The detailed trial plan is shown in Table 2.

**Table 2:** Trial visits schedule.

**Action visit**	**V0 screening**	**V1 BL**	**V2**	**V3**	**V4**	**V5**	**V6**	**V7**	**V8**	**V9**	**V10**	**V11**	**V12**	**V13**	**V14**	**V15**	**V16**	**V17**	**V18**	**V19**	**V20**	**V21**	**V22**	**V23**
Day (d)		1	2	3	4	5	8	9	10	11	12	15	16	17	18	19	22	23	24	25	26	45	90	180
Permitted delta in days (±) to d1	−42		0	0	+3	+3	+4	+4	+4	+4	+4	+4	+4	+4	+4	+4	+4	+4	+4	+4	+4	±3	±4	±5
		Treatment must be completed within 30 days, with a max. of 3 consecutive non-treatment days																						
Treatment IV 2 times daily		x	x	x	x	x	x	x	x	x	x	x	x	x	x	x	x	x	x	x	x			
Screening assessment																								
Patient information, informed consent	X																							
Inclusion/exclusion criteria	X																							
Demographics	X																							
Medical history	X																							
Diagnosis according to revised EEC	X																							
Physical examination	X																							
Randomization^#^	X^#^																							
Recurrent additional status data																								
Concomitant treatment history	X	X		X				X						X							X	X	X	X
Vital signs (pulse, BP)^**^	X	X	X	X	X	X	X	X	X	X	X	X	X	X	X	X	X	X	X	X	X	X	X	X
Height (only at screening), weight	X																				X		X	X
Endpoint assessment																								
ALSFRS-R		X																			X		X	X
Vital capacity (VC)^***^	X	X																			X		X	X
ALSAQ-5		X																			X		X	X
MUNIX		X^§^																			X		X	X
ECAS		X																			X		X	X
Safety assessment																								
Laboratory tests^^*^^,^^***^^	X	X		X				X						X							X	X	X	X
Pregnancy test^***^	X																							
Adverse events	X^****^	X	X	X	X	X	X	X	X	X	X	X	X	X	X	X	X	X	X	X	X	X	X	X

* *Laboratory tests: RBC, WBC, PLT, creatinine, GFR (MDRD), ALAT, ASAT, GGT, and CK, glucose*.

** *BP and pulse. On V1-V20 to be captured before and at 0, 10, 20, 30, 45, and 60 min after each administration of trial medication*.

*** *If not routine please perform only after informed consent. Pregnancy tests are to be repeated in case of suspicion and/or clinical signs of pregnancy during the course of the study participation*.

**** All Adverse Events must be collected from randomization. X

§ Baseline MUNIX analysis can be performed up to 2 weeks before V1. X

# *Randomization should be done only after all inclusion and exclusion criteria have been verified*.

### Outcomes

#### Primary Endpoints

Primary endpoints are safety and tolerability. The treatment with fasudil is considered safe for an individual patient if no drug-related serious adverse events (SAE) is recorded through to visit V23. The treatment with fasudil is considered tolerable if participants do not discontinue treatment due to suspected drug-related adverse events (AE). The proportions of patients for whom the treatment is tolerable/safe are derived for each treatment group.

#### Secondary Outcomes

Secondary endpoints are the survival time and the change of ALSFRS-R, ALSAQ-5, ECAS, MUNIX (18), and SVC from baseline to visits V20, V22 and V23. Secondary safety endpoint is safety until V20.

### Biosample Collection Plan

As part of the therapeutic trial we will collect biosamples which will be used for the analysis of pharmacodynamics, disease progression, genotyping and potentially for future studies. EDTA-blood, serum, plasma, urine, and saliva will be collected from all individuals at specified time points. At V20 (or exceptionally on V19 or V18), a CSF sample and a blood plasma sample will be collected to determine the peak concentration of fasudil and hydroxyfasudil as well as ROCK activity (Table 3). Individual alterations of the biosample collection and analysis plan will not be classified as violations of the general study protocol. All biomaterial samples will be stored at the Biobank of the University Medical Center of Göttingen, except for samples that will be analyzed immediately.

**Table 3:** Biosample collection plan.

**Action visit**	**V0 screening**	**V1 BL**	**V2**	**V3**	**V4**	**V5**	**V6**	**V7**	**V8**	**V9**	**V10**	**V11**	**V12**	**V13**	**V14**	**V15**	**V16**	**V17**	**V18**	**V19**	**V20**	**V21**	**V22**	**V23**
Day (d)		1	2	3	4	5	8	9	10	11	12	15	16	17	18	19	22	23	24	25	26	45	90	180
Permitted delta in days (±) to d1	−42		0	0	+3	+3	+4	+4	+4	+4	+4	+4	+4	+4	+4	+4	+4	+4	+4	+4	+4	±3	±4	±5
		Treatment must be completed within 30 days, with a max. of 3 consecutive non-treatment days																						
Treatment IV 2 times daily		x	x	x	x	x	x	x	x	x	x	x	x	x	x	x	x	x	x	x	x			
Biosample collection																								
EDTA-Blood (Genotyping)		X																						
CSF (e.g., Fasudil concentration, NfL)																			(X°)	(X°)	X°			
Plasma (e.g., Fasudil concentration and ROCK-activity)^***^	X	X																	(X°)	(X°)	X°			X
Serum (e.g., NfL, urate)		X																			X			X
Urine (e.g., soluble p75ECR)		X																			X			X
Saliva (e.g., Chromogranin A)		X																			X			X

*X° CSF (and concomitant plasma) analysis will be performed once 60 min after the end of trial drug infusion in the morning, regularly on V20 (exceptionally on V18 or V19). IV-Treatment must have taken place at least on the 2 days preceding CSF/plasma analysis*.

*** *If not routine please perform only after informed consent. All biomaterials must be taken directly after administration of the trial drug in the morning except CSF (see above, X°)*.

### Safety

During the study, the definitions of the directive 2001/20/EC are effective. All AE, irrespective of seriousness, will be collected from the day of randomization to the follow-up visit/end of trial visit at the time points specified in the trial visits schedule. The investigator should report any AE occurring in this time period that is believed to be related to study drug or protocol-specified procedure. All SAE for which a causal relationship with the administration of study medication may not be ruled out (serious adverse reactions, SAR) need to be defined and reported as suspected unexpected serious adverse reactions (SUSAR). Once an AE is detected, it should be followed until its resolution or stabilization and assessment should be made at each visit (or more frequently, if necessary) of any changes in severity, the suspected relationship to the study, the interventions required to treat it and the outcome. All SAE will be reviewed by an independent medical monitor, who will be independent from the reporting investigator, the trial sponsor and the coordinating investigator. The investigator must carry out a causality assessment for all AEs. The relationship of an AE to the study treatment regimen has to be recorded on the CRF and defined as not related, unlikely, possible related, probable related or highly probable/definite.

Expedited reporting applies if the AE is considered serious, unexpected, and drug related (SUSAR). This type of SAE must be reported by the CRO to the appropriate national health authority and ethics committees within 15 days; fatal or life-threatening events must be reported within 7 days. All person-related data will always be transmitted pseudonymized/de-identified. The CRO will immediately, within 15 days after it becomes known, report all circumstances that require a revision of the risk-benefit analysis to the relevant ethics committees and the federal authorities. This especially includes:

- Singular cases of expected SAE with an unexpected outcome.
- Increased incidence of expected SAE that are judged as being clinically relevant.
- SUSARs which occur after termination of the clinical trial (6 months) after termination or exclusion)
- Events related to study procedures or development of the study medication, which could affect a subject's safety.

The study will be monitored by an independent safety monitoring board (SMB), which will come together once before the start of the trial and then hold telephone conferences approximately every 3 months during the trial in order to review trial progress, safety data, and adherence to protocol, with the frequency of meetings depending on the rate of recruitment and safety issues.

### Data Recording and Study Monitoring

The documentation of the study data in adherence to the Good Clinical Practice (GCP)-guidelines and the clinical trial protocol is the responsibility of the local investigator. Original data (source documents) remain in hospital medical records and information on the eCRF must be traceable and consistent with the original data. Source documents are e.g., laboratory results, ALSFRS-R measurements, vital capacity measurements, and quality of life questionnaire. Original written informed consent signed by the patient is kept by the investigator and a signed copy will be given to the patient. No information in source documents about the identity of the patients will be disclosed. All data collected in this study must be entered in an eCRF which has to be completed by the investigator or authorized trial personnel and signed by the principle investigator. This also applies for those patients who do not complete the study. If a patient withdraws from the study, the reason must be recorded in the eCRF. The investigator is responsible for ensuring the accuracy, completeness, and timeliness of all data reported to the sponsor in the eCRFs and in all required reports.

After database lock, the principal investigator will receive data on an electronic device that includes the investigational site data for archiving in the Investigator Site File (ISF). Data are processed by data management of the CRO with the support of a study database (eCRF) according to the SOPs of the CRO. The evaluation of the data takes place by programmed validity and consistency checks.

Monitoring activities are performed to ensure that the trial is conducted in accordance with the trial protocol, the principles of GCP, and local legislation. A monitoring manual describing in detail the scope of the monitoring activities will be prepared. A monitoring visit report is prepared for each visit describing the progress of the clinical trial and all identified problems.

### Ethics and Dissemination

This trial will be conducted in accordance with the current ICH-GCP-guidelines. GCP is an international ethical and scientific quality standard for designing, conducting, recording and reporting trials that involve the participation of human subjects. Compliance with this standard provides public assurance that the rights, safety and well-being of trial subjects are protected and consistent with the principles that have their origin in the Declaration of Helsinki and that the clinical trial data are credible. Prior to study start the following documents will be obtained:

- Approval of ethics committees (lead and local ethics committees)
- Approval of national competent authorities: Bundesamt für Arzneimittel und Medizinprodukte (BfArM), Schweizerisches Heilmittelinstitut (SwissMedic), Agence nationale de sécurité du médicament et des produits de santé (ANSM)
- Notification to applicable regional authorities
- Informed Consent
- Insurance
- Data privacy and confidentiality.

Irrespective of study outcome, the results will be presented during scientific symposia and published on www.clinicaltrials.gov and in a peer-reviewed, international journal after written approval by the involved parties and respecting the privacy of the participants.

### Role of the Study Centers

In total, 16 recruiting study centers participate in the ROCK-ALS trial at the time of writing. In Germany, all centers (Berlin, Dresden, Essen, Göttingen, Halle (Saale), Hannover, Jena, Munich, Münster, Ulm, Würzburg) belong to the German Network for Motor Neuron Diseases (MND-NET) and represent specialized referral centers for motoneuron disorders. The Swiss center in St. Gallen is the largest ALS clinic in Switzerland. In France, four highly dedicated ALS referral centers participate in the trial (Marseille, Montpellier, Nice, Tours).

It is expected that each center

- randomizes 8–10 patients (in France and Switzerland 6 per center), administers the treatment, performs all required clinical examinations and takes scores, collects the biospecimens, and enters all data into the eCRF
- contributes at least one principle investigator, one clinical neurologist as study physician and one study nurse dedicated to the trial
- trains sufficient personnel to perform MUNIX assessments.

In addition, an associated center in Warsaw (without explicit funding through E-Rare and thus outside of the formal trial protocol) will recruit patients with the same inclusion and exclusion criteria for a biosample collection, but without treatment.

Safety analyses will be performed at the local laboratories at each center. Drug levels (riluzole, fasudil, and hydroxyfasudil) will be determined at the Medizinische Labor Bremen (MLB). Multiple other research labs are foreseen for the evaluation of liquid biomarkers (e.g., neurofilament, soluble p75^ECR^, chromogranin A, and urate). In addition, we will attempt to genotype each patient by whole genome sequencing, which will be performed in collaboration with the CReATe Consortium.

The trial will be supported and monitored by the Munich Trial Center (MSZ), an academic CRO with long-standing experience in the conduct of clinical trials, particularly IITs.

### Statistical Analysis

#### Sample Size Calculation

A sample size of 102 patients (i.e., 34 patients per treatment group) yields sufficiently narrow confidence intervals for the difference in proportions between the placebo group and the treatment groups for both primary endpoints, the proportion of patients without drug-related SAEs and the proportion of patients without treatment intolerability. Under the assumption of no difference between the treatment groups and the placebo group the half-width of the 95% confidence interval for the difference in proportions is at most 0.24. Expected are high proportions of tolerability and safety in which case the confidence interval becomes narrower. If the proportion is for instance 0.9 the half-width reduces to 0.14, which is considered sufficiently narrow. Calculations were carried out using the software nQuery 4.0. Adjusting for dropout of 15% we aim to recruit a total number of 120 patients (i.e., 40 patients per treatment group).

#### Primary Analysis

The study will yield information about safety, tolerability, and efficacy. The main focus of the primary analysis is to determine whether either one or both doses are safe and tolerable. Each patient will be treated with fasudil or placebo over 45 min twice a day for 20 days. Significant drug intolerance will result in stopping the infusion and termination of the trial participation for this patient.

The proportions of patients for whom the treatment is tolerable/safe are derived for each treatment group. For both active treatment groups and for both, safety and tolerability, separately the difference in proportions to the placebo group will be calculated with its 95% confidence interval. Subsequent analyses will model tolerability and safety in logistic regressions adjusting for randomization stratification factors and important prognostic factors assessed at baseline. Treatment group differences will be reported in terms of odds ratios with 95% confidence intervals.

Primary analyses of safety and tolerability will be carried out on the ITT population. For the purpose of the tolerability analyses, subjects who discontinue during the treatment period will be considered as worst case, i.e., no drug tolerability. If a number of patients withdraw from the study following completion of the treatment period, there observations will be dealt with as independent right censoring and the time to first drug-related SAE will be considered as primary endpoint. The difference in proportions free of any drug-related SAE at V23 will be assessed using Kaplan-Meier estimates and corresponding 95% confidence intervals. Subsequent analyses will model time to first drug-related SAE in Cox proportional hazard regressions adjusting for randomization stratification factors and important prognostic factors assessed at baseline. Treatment group differences will be reported in terms of hazard ratios with 95% confidence intervals. The ITT analyses will be complemented with a per-protocol (PP) analysis. Differences between both analyses will be reported and evaluated in detail.

#### Secondary Analysis

##### Efficacy

Efficacy outcomes including ALSFRS-R, ALSAQ-5, SVC, and MUNIX through to visit V23 will be analyzed by means of Gaussian linear model for repeated measures (so-called MMRM) with treatment group, time (visits V20, V22, V23), treatment-by-time interaction, region, and stratum of onset as factors and baseline measurements of the outcome as covariate. The error terms are assumed to follow a multivariate normal distribution with unstructured covariance. Least squares mean changes from baseline will be reported for the treatment groups with 95% confidence interval (CI) as well as the difference between the least squares treatment group means with 95% CI and *p*-value testing the null hypothesis of no treatment effect. The analysis will be primarily performed on the ITT population, but complemented by PP analysis.

Survival time will be used as a secondary endpoint. The Kaplan-Meier method will be applied to estimate the survival probabilities in each group. The 95% CIs will be calculated with the variance derived according to Greenwoods' formula. Subjects will be right-censored at end of their follow-up. Pairwise group comparisons against placebo will be analyzed using exact log rank tests. Cox proportional hazards regression will be carried out if there are a sufficient number of events.

## Discussion

### Choice of Target and Drug

Disease-modification is an urgent need for the treatment of ALS. Although a multitude of different pathomechanisms are thought to contribute to disease progression, only the glutamate antagonist riluzole and the antioxidant edaravone have been approved so far, both with limited therapeutic potential. Novel drugs should thus target different, preferentially yet unaddressed mechanisms and ideally act in an additive or even synergistic manner with both licensed substances. ROCK inhibition appears as a promising novel approach in this respect acting on multiple pathomechanisms: it has been shown to increase axonal regeneration, attenuate neuronal cell death, modulate microglial activity and beneficially affect survival and motor function in mouse models of ALS. Several ROCK inhibitors with different chemical backbones have been developed (19), but only fasudil and ripasudil have been licensed for clinical use so far. Ripasudil has only been licensed as local treatment for glaucoma, but fasudil is used as systemic drug for intravenous application and is therefore the ROCK inhibitor of choice employed in this trial.

### Choice of Trial Type and Design

Because of the long-standing clinical use of fasudil for the treatment of subarachnoid hemorrhage-induced vasospasms, a phase I dose-escalation trial is not required. However, since there are no published data on safety and tolerability of fasudil in ALS patients, a phase IIa trial has been designed. Because the drug is only licensed as IV formulation and the maximally approved dosage is 1.260 mg over 14 days (3 × 30 mg per day), a compromise in the treatment duration was made: the treatment duration of 20 days (until V20) is long enough to estimate safety and tolerability of fasudil IV in ALS patients and to expect a long-lasting regulation of motoneuron survival pathways and alteration of microglial activity. Long-lasting effects of ROCK inhibition have been observed after a circumscribed drug application in animal models (20). At the same time, 20 treatment days are short enough to expect reasonable patient adherence for an IV therapy and to keep trial costs in a financially feasible range.

Results of this phase IIa study will allow an estimation if a longer treatment period could be favorable and if an oral formulation (e.g., with extended release tablets) can be implemented for subsequent phase IIb and phase III studies. Insufficient tolerance or significant safety concerns after application of fasudil IV in both doses will argue against a subsequent phase IIb or III study. On the other hand, lack of significantly improved efficacy readouts will *per se* not argue against a follow-up study, because we can only estimate the magnitude of improvement at this stage and the study may be underpowered to detect a significant improvement.

In order to achieve a long-lasting disease-modifying effect in a chronic neurodegenerative disorder, such as ALS, a future treatment most likely will have to be administered in a continuous way, which is best achieved by an oral medication. In the case of satisfactory tolerance and safety of IV fasudil in ALS patients, we plan to propose a follow-up phase IIb study (extended dose-finding) exploring the safety, tolerability and clinical effects of oral fasudil over 52 weeks in three different dosages. Subsequently, an international, multi-site, randomized phase III study will have to be performed aiming at an efficacy readout in a larger trial population.

Concomitant treatment with riluzole is considered standard therapy; in order to reduce bias, patients need to be treated with riluzole for at least 4 weeks. Most ALS patients in the participating countries receive riluzole and in most participating countries it is the only licensed drug, which therefore will not be withheld. For ethical reasons, patients who have started on edaravone therapy shall continue edaravone treatment. Edaravone treatment must not be discontinued for reasons of trial participation.

### Risks and Benefits

Fasudil is approved for the treatment of vasospasms following subarachnoid hemorrhage by Japanese authorities since 1995. Several thousand patients have been treated with this drug and thus the safety profile of fasudil in humans is well-known. In patients with ischemic stroke, fasudil also showed a beneficial effect on functional outcome and was well-tolerated in the study population (10). Numerous clinical trials for other disease conditions, mainly cardiovascular disorders, have been performed and did not raise major safety issues. Therefore, it is considered justified and safe to assess the effect of fasudil in patients with ALS.

The administration of placebo in our study population is acceptable since both placebo and fasudil are given as add-on to the standard therapy riluzole, which is an inclusion criterion. If a treatment with edaravone has been started prior the trial, it should be continued throughout the study.

A direct interaction of the two substances fasudil and riluzole leading to adverse effects has not been described yet and is not expected based on their different mechanism of action. Research in www.drugbank.ca (global drug properties), http://dgidb.genome.wustl.edu/ (drug-gene interactions), http://medicine.iupui.edu/clinpharm/ddis/index (Cytochrome P450 drug interaction table), and http://lmmd.ecust.edu.cn:8000 (chemical ADMET properties) did not reveal any known or expected drug interactions on the enzymatic or genetic level. To detect a putative influence of fasudil on riluzole levels, riluzole levels will be assessed.

In addition to its neuroprotective and pro-regenerative effects, the kinase inhibitor fasudil was also shown to affect microglial activity. The mechanism of action of riluzole is thought to be in part related to its stabilizing effect on sodium channels and the resulting reduction of presynaptic glutamate release. It can thus be hypothesized that the neuroprotective effect of riluzole—the standard therapy for patients with ALS—could be increased by the neuroprotective effect of fasudil in an additive manner.

Hemorrhage, particularly intracerebral bleedings, are described as a side effect of fasudil. This, however, was assessed in a population of patients with SAH and the incidence of hemorrhage was not significantly different in the placebo-treated group. Thus, hemorrhage is not expected to be a risk for our patient population. Moreover, patients with a personal or family history of intracranial bleeding, known intracerebral aneurysms or Moyamoya disease will be excluded from the trial.

Irrespective of the trial treatment longtime benefit may be limited, yet cannot be ruled out. Furthermore, all patients will contribute to the generation of valid data from a prospective, placebo-controlled clinical study. In addition, exploratory assessments will help to identify markers that indicate prognosis and activity of disease.

In conclusion, the ROCK-ALS trial will yield important data on safety, tolerability, and efficacy of a novel therapeutic strategy, ROCK inhibition, as disease-modifying treatment of ALS. The biomarker collection linked to this trial will provide additional information on target engagement and the usefulness of selected molecular markers as indicators of progression.

## Author Contributions

PL and JK developed the trial concept, wrote the trial protocol, wrote and edited the manuscript. MW, WC, CN, MK-K, MBe, HB, CB, RF, and ALu provided input on trial design, and critically edited the manuscript for content. TF, RH, and ALe detailed the statistical aspects of the study in the study protocol, and critically reviewed the manuscript. RG contributed to the preclinical data. SA, MBä, MBoe, NB, IC, PC, MD, TG, JG, RG, AH, JK, TL, FM, TM, SP, YR, JeS, JoS, M-HS, JS, JW, and EZ contributed to and corrected the manuscript, actively participating study center. All named authors meet the International Committee of Medical Journal Editors (ICMJE) criteria for authorship for this article, take responsibility for the integrity of the work as a whole, and have given their approval for this version to be published.

### Conflict of Interest Statement

As potential conflict of interest, we report that PL (together with Lars Tönges) are inventors on a patent held by the University Medical Center of Göttingen on the use of fasudil for the treatment of ALS (EP 2825175, US 9980972B2). The remaining authors declare that the research was conducted in the absence of any commercial or financial relationships that could be construed as a potential conflict of interest.
